# Detecting SARS-CoV-2 lineages and mutational load in municipal wastewater and a use-case in the metropolitan area of Thessaloniki, Greece

**DOI:** 10.1038/s41598-022-06625-6

**Published:** 2022-02-17

**Authors:** Nikolaos Pechlivanis, Maria Tsagiopoulou, Maria Christina Maniou, Anastasis Togkousidis, Evangelia Mouchtaropoulou, Taxiarchis Chassalevris, Serafeim C. Chaintoutis, Maria Petala, Margaritis Kostoglou, Thodoris Karapantsios, Stamatia Laidou, Elisavet Vlachonikola, Anastasia Chatzidimitriou, Agis Papadopoulos, Nikolaos Papaioannou, Chrysostomos I. Dovas, Anagnostis Argiriou, Fotis Psomopoulos

**Affiliations:** 1 Institute of Applied Biosciences, Centre of Research and Technology Hellas, Thermi, 57001 Thessaloníki, Greece; 2 Department of Genetics, Development and Molecular Biology, School of Biology, Aristotle University of Thessaloniki, 54124 Thessaloníki, Greece; 3 School of Veterinary Medicine, Aristotle University of Thessaloniki, 54124 Thessaloníki, Greece; 4 Department of Civil Engineering, Aristotle University of Thessaloniki, 54124 Thessaloníki, Greece; 5 Department of Chemistry, Aristotle University of Thessaloniki, 54124 Thessaloníki, Greece; 6 EYATH S.A., Thessaloniki Water Supply and Sewerage Company S.A., 54636 Thessaloníki, Greece; 7 Department of Food Science and Nutrition, University of the Aegean, Myrina, 81400 Lemnos, Greece

**Keywords:** Computational biology and bioinformatics, High-throughput screening, Software

## Abstract

The COVID-19 pandemic represents an unprecedented global crisis necessitating novel approaches for, amongst others, early detection of emerging variants relating to the evolution and spread of the virus. Recently, the detection of SARS-CoV-2 RNA in wastewater has emerged as a useful tool to monitor the prevalence of the virus in the community. Here, we propose a novel methodology, called **lineagespot**, for the monitoring of mutations and the detection of SARS-CoV-2 lineages in wastewater samples using next-generation sequencing (NGS). Our proposed method was tested and evaluated using NGS data produced by the sequencing of 14 wastewater samples from the municipality of Thessaloniki, Greece, covering a 6-month period. The results showed the presence of SARS-CoV-2 variants in wastewater data. **lineagespot** was able to record the evolution and rapid domination of the Alpha variant (B.1.1.7) in the community, and allowed the correlation between the mutations evident through our approach and the mutations observed in patients from the same area and time periods. **lineagespot** is an open-source tool, implemented in R, and is freely available on GitHub and registered on bio.tools.

## Introduction

Nearly a year after the first report of SARS-CoV-2 in Wuhan, China, the virus has spread at an unprecedented pace causing a global pandemic. The predominant transmission process of SARS-CoV-2 is through droplets and contact between people, and whether a person is infected can be identified by rapid molecular strategies. SARS-CoV-2 genotyping for epidemiological investigations/to understand its molecular epidemiology relies on PCR-based methods, affordable for most laboratories. However, these methods are not easily scalable, especially in large urban areas, where a high number of individuals have to be tested to assess virus and variant spread among the population, or when new variant/mutation screening is needed, which can be attained only by sequencing methods. Interestingly, the viral RNA can also be detected in wastewater, with SARS-CoV-2 RNA levels in wastewater correlating with the COVID-19 epidemiology^1–3^. Indeed, in the previous work of Petala et al.^3^. normalized viral copy levels in Thessaloniki wastewater agreed with the epidemiological conditions in the city. Thessaloniki is the second largest city in Greece with around 1 million inhabitants. The city is a chief gateway for entrepreneurs, traders, university students and tourists visiting Greece and, as such, it was the place where the Greek patient “zero” firstly appeared in March 2020 as well as the mutation characteristic for the Beta variant which was first detected in South Africa. In other words, the city of Thessaloniki is a suitable place as a case study for identifying new mutations in the city wastewater before scattering to the rest of the country.

The presence of SARS CoV-2 RNA in wastewater provides us with a unique opportunity, i.e., to identify the most prevalent virus lineages through the analysis of the traces evident in wastewater samples. Since the beginning of the pandemic a lot of countries have been studying SARS-CoV-2 mutations in wastewater for COVID-19 surveillance^4–9^. Yet, it still remains an open issue as there are no widely accepted methods that can sufficiently address this. The most commonly used approaches involve the sequencing of the wastewater samples, and the consequent application of low frequency mutation analysis methods^10^ or metagenomic approaches^11,12^. In either case, the interpretation of the results focuses on the detection of specific mutations^10^ or lineages^2^ such as the Alpha (B.1.1.7) and Beta (B.1.351) variants or new uncharacterized mutations^12^.

In this work we propose a novel methodology called ***lineagespot***, implemented as a software tool that can facilitate the detection of SARS-CoV-2 lineages in wastewater samples using next-generation sequencing (NGS). The method is tested and validated across 14 municipal wastewater samples retrieved in Thessaloniki, Greece in 14 different time periods, and correlated with the mutations and lineages observed in patients from the same area time points. Based on a variation of the Illumina ARTIC pipeline for the identification of mutations at low frequencies (< 0.01), and the lineage assignments defined by Pangolin^13^, this method identifies all SARS-CoV-2 variants present in the wastewater, and attempts to infer the potential distribution of the SARS-CoV-2 lineages. The methodology is proven to be effective in detecting the mutational load in the wastewater, with the inferred lineages being roughly aligned to the predominant lineages identified through targeted patient-derived genotypes.

## Results

All acronyms and abbreviation used in the text are explained in Table 7.

### Comparison of variant calling methods reveals freebayes as the best-performing tool for low-frequency variant detection

The proposed methodology was initially applied on a selected wastewater sample (corresponding to the 05–11 February 2021 time period), using NC_045512 as the reference genome, and for which three different variant callers were assessed: (1) *freebayes*^14^, (2) *mpileup*^15^ and (3) *GATK Mutect2*^16^ (cancer only mode). In terms of parameters, *freebayes* was used with a low mutation frequency parameter of 0.01, *mpileup* reported every position (either reference, or mutation), and *GATK Mutect2* was used with the default parameters. The full bioinformatic pipeline used to identify the mutations is described in the “Raw data analysis” Section. Additionally, the proposed variant calling pipeline (with all set parameters) is also provided in the [lineagespot GitHub repository] (https://github.com/BiodataAnalysisGroup/lineagespot/blob/master/inst/scripts/raw-data-analysis.md).

An example of the output produced by the comparison, is shown in Table 1. In this table the number of common mutations (N_t_) between the lineage definition (retrieved either as a Pangolin rule or through the outbreak.info definition) and the mutations of the input dataset is captured for each lineage.

**Table 1:** Each row in the table (used as an internal structure) corresponds to a single lineage.

Lineage	Total number of lineage’s characteristic mutations	N_t_^freebayes^	N_t_^mpileup^	N_t_^gatk^
*B.1.177*	17	09	07	07
*B.1.1.7*	21	10	15	4
*B.1.351*	19	05	08	08

The columns correspond to the different number of SARS-CoV-2 mutations (N_t_) that are captured in the sample.

The number of common mutations are compared pairwise for the three variant callers. For each lineage, the absolute values of the differences between the N_t_ metrics are calculated. As an example, for lineage *B.1.1.7*, the output produced by using the *freebayes* variant caller tool in the first step of the methodology, returns ${N}_{t}=10$ mutations satisfied, while the output of the *GATK Mutect2* tool returns ${N}_{t}=4$ mutations satisfied. As a result, the two outputs exhibit a difference of $\left|{N}_{t}^{freebayes}-{N}_{t}^{gatk}\right|=6$ (Table 2).

**Table 2:** Difference between the number of common mutations across the three variant callers.

Lineage	|N_t_^freebayes^ − N_t_^gatk^|	|N_t_^freebayes^ − N_t_^mpileup^|	|N_t_^gatk^ − N_t_^mpileup^|
*B.1.177*	2	2	0
*B.1.1.7*	6	5	11
*B.1.351*	3	3	0

**Table 3:** Summary table of the three output files produced by *freebayes*, *mpileup* and *GATK mutect2* variant caller.

Variant calling comparison	Number of differences	Max absolute N_t_ difference
*freebayes–gatk*	3791	31
*freebayes–mpileup*	3140	33
*gatk–mpileup*	1571	31

The three variant callers are compared in pairs.

**Figure 1:**
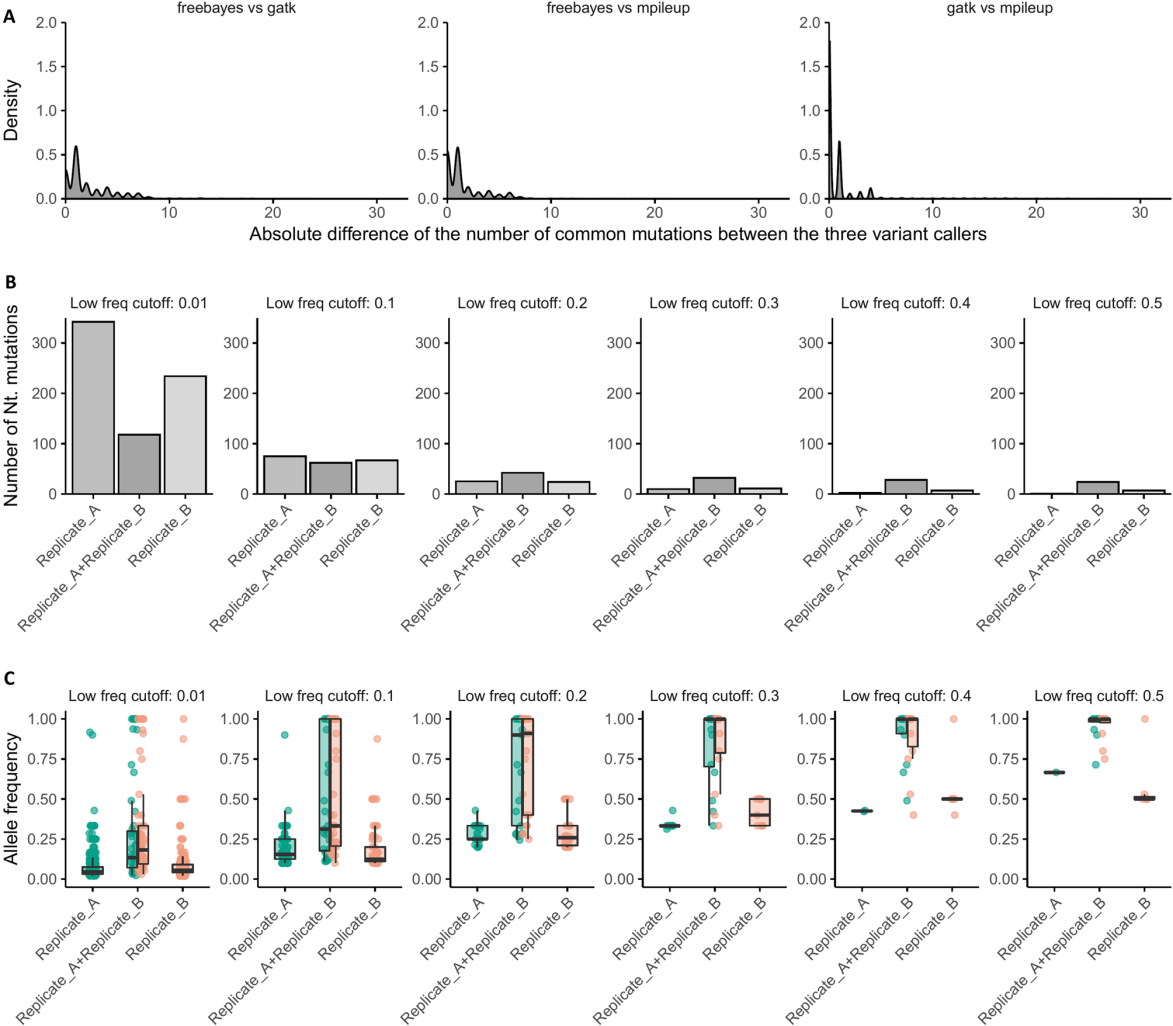
Evolution of mutations across different low frequency parameters. (**A**) Density plot of the absolute ${N}_{t}$ difference values between the number of common mutations of the three variant calling tools used (pairwise comparisons). (**B**) Number of reads for each replicate and for the common mutations. (**C**) The corresponding allele frequency for each replicate and for the common mutations.

In addition, Table 2 gathers the differences between the number of common mutations across the three variant callers, which are consequently used for their overall comparison (Figure 1A). In Table 3, a summary of the differences is provided containing the total number of differences for the compared lineages and the maximum ${N}_{t}$ differences for each pair of files.

Based on the above comparison, we consider that the most productive and informative approach is to utilize *freebayes* as the variant calling tool. The rest of the results shown below, are based only on the *freebayes* tool output.

### Evaluating lineage-specific mutations across time periods

#### Sensitivity

In order to investigate how specific amino acid substitutions evolve over time, all VCF files were merged into a combined table in which all the detected nucleotide mutations along with the corresponding amino acid substitutions that have been identified are stored. Moreover, information about the allele frequency, and the overlapping gene is also provided for each mutation. Table 4 gives an example of the overall information.

**Table 4:** Snapshot of table containing every mutation per sample along with the corresponding gene and the amino acid substitutions.

CHROM	POS	REF	ALT	DP	AD alt	Gene name	HGVS	AF	Sample
NC_045512.2	326	T	A	7	1	ORF1ab	T21I	0.143	Sample A
NC_045512.2	378	T	C	10	1	ORF1ab	V38A	0.100	Sample A
NC_045512.2	408	A	T	10	1	ORF1ab	D48V	0.100	Sample B
NC_045512.2	433	T	C	10	2	ORF1ab	V56V	0.200	Sample C
NC_045512.2	442	C	T	10	1	ORF1ab	G59G	0.100	Sample C

Having a structured format, we first examined if the number of reads that were produced during PCR amplification is affecting the number of mutations that are identified in every sample. For this reason, two replicates of the same biological specimen were produced. The first replicate (*Replicate A*) contained 181,880 reads while the second replicate (*Replicate B*) contained 69,706 reads. The two replicates were analyzed as described in the “Raw data analysis” section and two VCF files were produced which contained 401 and 293 mutations respectively; of these, 59 mutations were common in both replicates, 342 mutations were unique for Replicate A and 234 unique for Replicate B.

A Student's t-test was performed in order to compare the mean values of the allele frequency between the two replicates and an F test to compare the variances. All tests resulted in p values higher than the 0.05 threshold, meaning that no statistically significant difference was found between the two replicates. On the contrary, when comparing two samples coming from different time periods there was a significant difference on the mutations’ allele frequency (p values from Student’s t-test and F test were below 0.05).

Moreover, for the same replicates, 6 different pairs of VCF files were generated in order to investigate how the number of mutations located changes while increasing the low mutation frequency parameter of 0.01 that was used at *freebayes* tool during the raw data analysis. The thresholds that were chosen were *0.01*, *0.1*, *0.2*, *0.3*, *0.4* and *0.5*. In Fig. 1B, the evolution of the number of mutations found is shown while Fig. 1C gives the corresponding allele frequency.

### Mutational load detection

The proposed methodology was applied on the wastewater samples, across 14 time periods. Information regarding the total number of reads, the number of reads mapping to SARS-CoV-2 reference genome etc. are provided in Supplementary Table 1.

**Figure 2:**
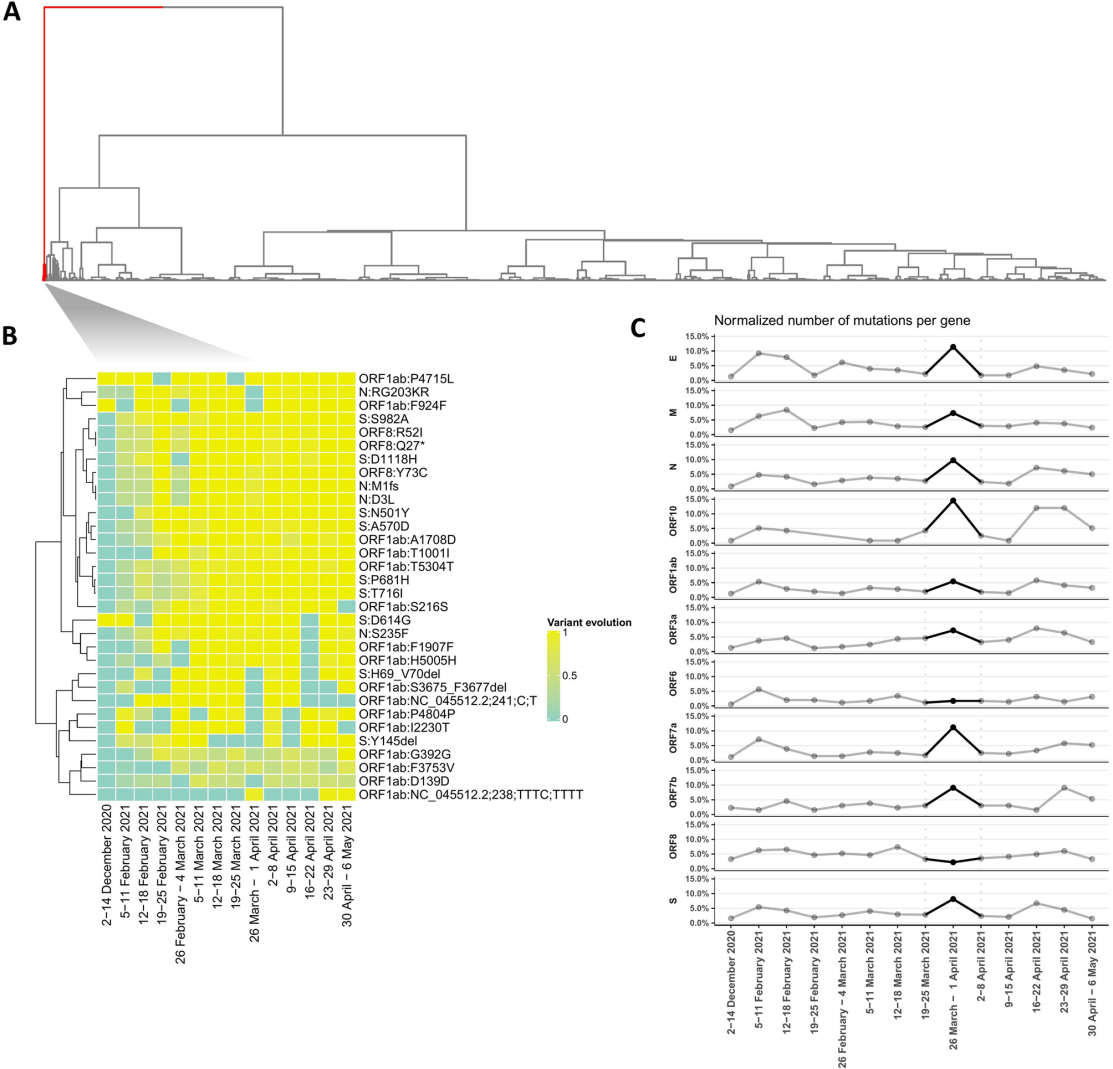
Unsupervised mutation clustering was performed on a table containing all amino acid substitutions (**A**) Hierarchical clustering shows the clustered collapsed amino acid substitution using the Euclidean distance as a distance metric and ward.D as a clustering method. (**B**) Hierarchical clustering based on the cluster 1 of the (**A**). The heatmap shows the mutation evolution across the different periods. (**C**) Number of mutations per gene across the different periods. The values of the plot were normalized based on the length of each gene.

To this end, Table 4 was collapsed at the gene and amino acid substitution level, therefore reducing any data noise that is introduced by nucleotide mutations that correspond to the same amino acid change. In Fig. 2A all collapsed amino acid substitutions were clustered accordingly based on Euclidean distance, while Fig. 2B highlights specific mutations (cluster 1) that exhibit significant difference in behavior.

Moreover, we studied the number of amino acid substitutions per gene over each time period. In Fig. 2C the number of mutations were clustered based on Euclidean distance, after all count values were normalized by each gene’s length.

### Detection of variants of concern/variants of interest

**Figure 3:**
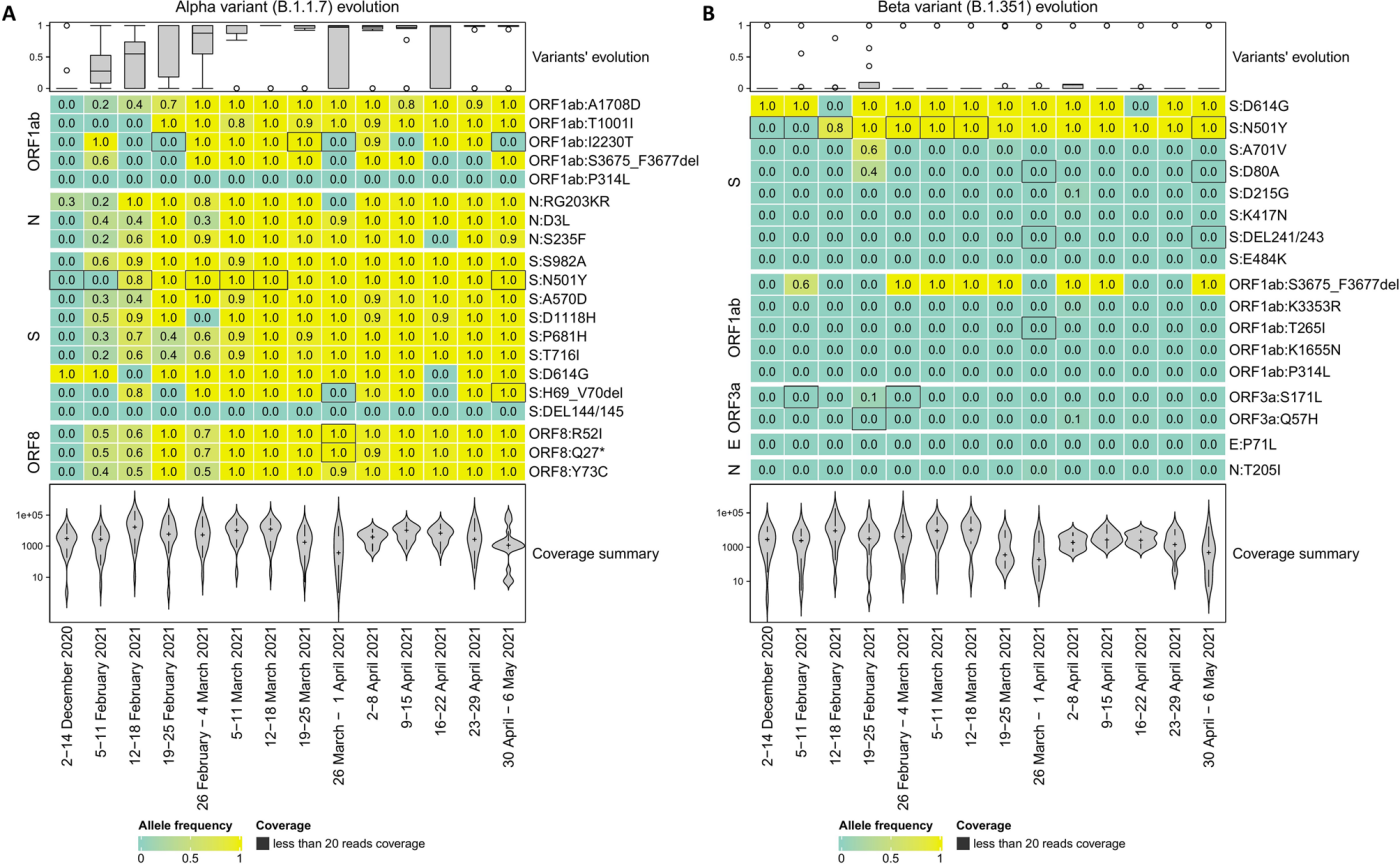
Clustering amino acid substitutions for the Alpha (B.1.1.7) and the Beta (B.1.351) variants. Heatmap displays the corresponding allele frequency (AF) of each period per amino acid substitution. (**A**) Evolution of B.1.1.7-detected mutations. (**B**) Evolution of B.1.351-detected mutations. Positions with low coverage (less than 20 reads) are depicted with dark gray color.

Furthermore, the evolution of lineage-specific mutations over the 14 time periods was studied. To this end, two Variants of Concern were selected; the Alpha (B.1.1.7) and the Beta (B.1.351) variants which are shown in Fig. 3.

Moreover, we examined the prevalence rate of the two variants of concern by calculating the average allele frequency of their mutations. The results for the 14 time periods are presented in Table 5 and show that it does not lead to 100% sum per time period. The latter is caused due to overlapping mutations among the lineages and implies that the average value of all the mutations is not a reliable metric to characterize a specific lineage’s presence.

**Table 5:** Allele frequency metrics computed for the comparison with the clinical data.

	Average allele frequency of variants’ mutations		Average allele frequency of the unique variants’ mutations		Minimum allele frequency of the present (non-zero) unique variant’s mutations	
	*B.1.1.7* (%)	*B.1.351* (%)	*B.1.1.7* (%)	*B.1.351* (%)	*B.1.1.7* (%)	*B.1.351* (%)
2–14 Dec. 2020	0.00	0.00	0.00	0.00	0.00	0.00
5–11 Feb. 2021	32.71	0.00	35.15	0.00	16.67	0.00
12–18 Feb. 2021	51.92	9.05	49.20	0.21	39.53	1.47
19–25 Feb. 2021	79.78	22.15	85.84	14.20	58.06	35.48
26 Feb.–4 Mar. 2021	78.18	11.11	77.51	0.00	31.88	0.00
5–11 Mar. 2021	95.54	11.11	96.28	0.00	76.71	0.00
12–18 Mar. 2021	94.12	11.11	92.31	0.00	100.0	0.00
19–25 Mar. 2021	92.58	11.41	91.14	0.56	92.75	3.92
26 Mar.–1 Apr. 2021	74.09	11.51	74.27	0.63	89.49	4.44
2–8 Apr. 2021	96.89	12.30	95.98	1.56	92.14	2.03
9–15 Apr. 2021	86.20	11.10	81.99	0.00	77.15	0.00
16–22 Apr. 2021	74.32	10.97	74.50	0.00	89.22	0.00
23–29 Apr. 2021	81.60	11.11	83.81	0.00	93.41	0.00
30 Apr.–6 May 2021	93.54	11.11	91.55%	0.00	93.85	0.00

To this end the average allele frequency of all mutations, the average allele frequency of the unique mutations and the minimum allele frequency were calculated for each time period. Three metrics were used to quantify the presence of each lineage. Firstly, the “Average allele frequency of the mutation” which is the average allele frequency of all amino acid mutation of a lineage, the “Average allele frequency of the unique mutation” which is the average allele frequency of the unique (no shared with another lineage) amino acids mutations of a lineage, and finally the “Minimum allele frequency of the present (non-zero) unique mutation” which is the minimum allele frequency of the non-zero unique mutation of lineage.

Further in the analysis, we attempted to examine the minimal lineage support per time period. In this step we calculated the average allele frequency of the mutations unique to each variant of concern and produced a table which is more comparable with the clinical data metric. In this context, the minimum allele frequency of the present (greater than zero) mutations indicates the level of our confidence. The results of the two metrics are shown in Table 5.

### Assessing lineage assignment

Qualitative assessment between the major lineages found in the targeted clinical samples, retrieved from the ENA study ID: PRJEB44141 (ERP128154), and across the 14 time periods. The clinical samples (specific ENA entries listed in Supplementary Information file) exhibit the lineage distribution shown in Supplementary Table 2.

**Figure 4:**
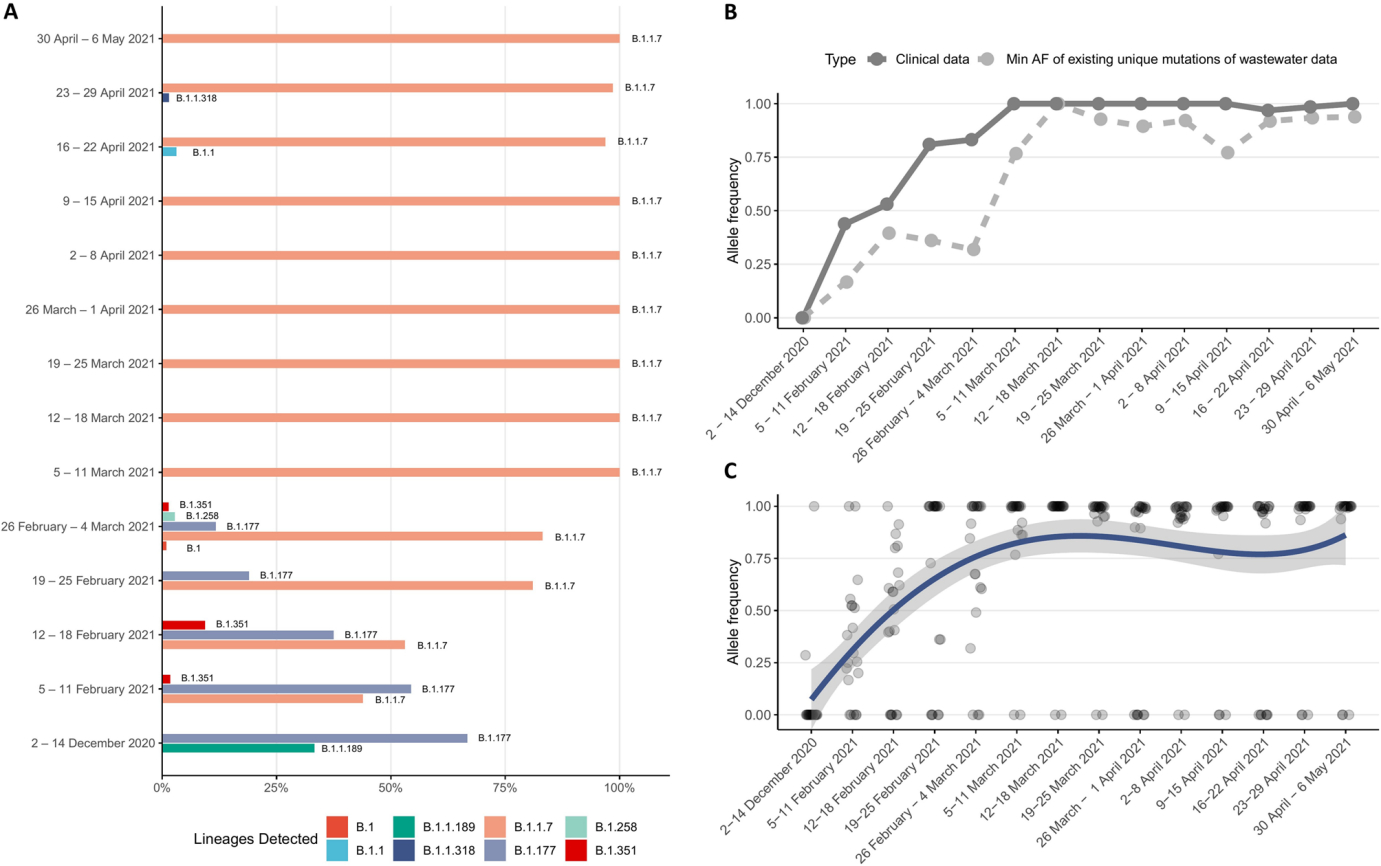
Comparison between wastewater samples and clinical data. (**A**) SARS-CoV-2 lineages detected on clinical samples over all time periods. (**B**) The percentage of presence of the Alpha (B.1.1.7) variant of concern (VoC) in the clinical samples and the estimated minimum level of presence of the same VoC in the wastewater data. (**C**) Average percentage of the presence of each characteristic mutation of the B.1.1.7 variant of concern. The line corresponds to the average value per time period. Mutations that are not found in a particular time point are detected in the neighbouring ones, thus leading to the variations in the average.

Based on the clinical results, as derived from targeted and non-randomized sampling of the Thessaloniki area, we can perform a direct comparison between the level of presence in a particular variant of concern (Alpha variant—B.1.1.7) in clinical and wastewater data (Fig. 4).

## Discussion

Analyzing wastewater, i.e. used water that goes through the drainage system to a treatment facility, is a way that researchers and surveillance systems can track pathogens, such as SARS-CoV-2, or biomarkers that are excreted in urine or feces. Monitoring effluents could be a reliable and more effective tool to estimate SARS-CoV-2 spread compared to sampling and testing the population, because wastewater surveillance can account for those who have not been tested and have only mild or no symptoms. Moreover, an effective and reliable methodology able to detect viral load and SARS-CoV-2 variants from municipal wastewater samples could decrease the cost of virus variant detection in the general population based on whole genome sequencing, since only a few samples must be processed and analyzed.

In this manuscript we present and validate a methodology named ***lineagespot***, making use of NGS data, able to detect lineages and mutational load of SARS-CoV-2. The methodology aims to aid the epidemiological system for the monitoring of COVID-19 pandemic in urban areas.

The method has been tested in different time point samples taken from the main Municipal Wastewater Treatment Plant of Thessaloniki—Greece, where effluents from approx. 750,000 inhabitants are collected. The ***lineagespot*** method demonstrated to be sensitive enough to identify and quantify differences in the mutational load, across various time points and was capable of recording the evolution of the B.1.1.7 lineage in the community. The method is capable of identifying SARS-CoV-2 mutations and characterize the abundance of already defined variants (Alpha, Beta variant etc.) within the same sample, assuming that mutations detected in short reads are within that set of mutations already sequenced. In addition, ***lineagespot*** allows the identification of amino acid substitutions which are not linked with any variant of concern but are dominant in particular time periods in a specific area. Moreover, the quantitative data obtained using ***lineagespot*** are in accordance with the trends of well-known mutations (such as D614G) in the same period with the overall epidemiological status of the municipal area. The application of ***lineagespot*** in such complex samples, like those from Wastewater Treatment Plants, was able to assign lineages and in agreement with the trend of the major lineages detected in the area of Thessaloniki, in the 14 time points by whole genome sequencing of samples from the general population.

Overall, the method developed herein was proven superior compared to other methodologies (Sanger sequencing)^4,7^, since it was more informative and sensitive enough to detect mutations with low frequency and able to assign with good approximation the correct lineage present in the municipality.

## Methods

### Sampling and isolation

Wastewater samples were collected from the entrance of the main Municipal Wastewater Treatment Plant of the city which accommodates sewerage of about 750,000 inhabitants. Wastewater entering this plant refers exclusively to citizens from urban districts of the city. Typical values of certain physicochemical properties of wastewater samples tested in this study are displayed in Table 6. These properties demonstrate, among others, the existence of suspended solids, dissolved organic matter, dissolved oxygen and salinity that may have strong impact on viral adsorption and decay because of oxidation and increased metabolic activity of microorganisms in wastewater. The residence time of wastewater until the entrance of the Plant is between 2 and 7 h (depending on the area), which is more than enough to allow viral adsorption and decay. Identification of mutations may be hindered by viral adsorption and decay and for this reason the present effort is particularly significant.

**Table 6:** Main quality characteristics of wastewater samples.

	pH	Electrical conductivity (S/cm)	Total suspended solids (mg/L)	BOD_5_ (mg/L)	COD (mg/L)	Dissolved organic carbon (mg/L)	UV absorption at 254 nm (1/cm)	Total nitrogen (mg/L)	Ammonium nitrogen (mg/L)	Total phosphorus (mg/L)	Copies/μL
02–14 Dec. 2021	7.5	8.5	620	385	960	35	0.35	62	28.5	11.5	36
05–11 Feb. 2021	7.8	9.6	930	525	1250	49	0.4	76	33	11.5	68
12–18 Feb. 2021	7.8	4.6	1200	650	1570	44	0.45	95	38	12	53
19–25 Feb. 2021	7.9	3.5	1225	684	1610	56	0.49	95	38.2	15.2	82
26 Feb.–4 Mar. 2021	7.8	2.9	1225	535	1383	53.5	0.47	78.5	36.7	12.4	179
5–11 Mar. 2021	7.6	2.8	1017	540	1373	50.2	0.47	71.7	36.8	12.1	102
12–18 Mar. 2021	7.8	4.5	852	580	1285	66.3	0.48	76.4	37.4	11.7	277
19–25 Mar. 2021	7.6	4.1	926	582	1467	60.8	0.48	79.3	39.6	11.6	467
26 Mar.–3 Apr. 2021	7.6	3.4	1095	660	1708	52.1	0.44	85.9	41.4	12.3	494
2–08 Apr. 2021	7.6	4.2	1054	667	1537	52	0.48	88.1	40.5	13.2	498
09–15 Apr. 2021	7.7	4.1	1025	579	1464	55.6	0.5	80	32.1	11.4	505

Sampling and handling of the wastewater samples were performed according to Petala et al.^3^. Briefly, samples were obtained using a refrigerated autosampler (6712 Teledyne ISCO) programmed to deliver a 24-h composite sample by mixing consecutive half-hour samples. Samples were transported to the lab on ice and were processed immediately. Three 50-mL aliquots of each untreated wastewater sample were subjected to centrifugation at 4000×*g* for 30 min. Afterwards, a composite sample was obtained from supernatants and pH was adjusted to 4 using 2 M HCl solution. Then, three aliquots of 40 mL each, were filtered through respective 0.45-μm pore-size, 47-mm diameter electronegative membranes (HAWP04700; Merck Millipore, Ireland) for Sars-CoV-2 nucleocapsid binding. Each membrane filter was rolled into a Falcon 15-mL conical centrifuge tube with the top side facing inward, and was subjected to RNA extraction real-time RT-PCR and testing for SARS-CoV-2 quantification in a separate facility where no clinical samples are processed to exclude the possibility of contamination. For every round of wastewater samples, a blank negative water control was handled and processed by the same steps through the whole procedure.

#### RNA extraction and SARS-CoV-2 quantification

Each electronegative membrane was subjected to RNA extraction process based on a phenol–chloroform-method^17^ coupled with magnetic bead binding. The following reagents were added sequentially: (a) 900 μL of guanidinium isothiocyanate-based “Lysis buffer I” [5 M guanidinium isothiocyanate, 25 mM EDTA, 25 mM sodium citrate (pH 6.2), 25 mM phosphate buffer (pH 6.4)] containing 1% N-Lauroylsarcosine, 2% Triton X-100, 2% CTAB and 2% PVP, (b) 18 μL β-mercaptoethanol, (c) 300 μL H_2_O. Tubes were mixed thoroughly by inversion and incubated at 4 °C on horizontal rotator (50 rpm) for 10–30 min. Subsequently, 1.2 mL of “Lysis buffer II” [prepared by mixing 152.5 gr guanidinium hydrochloride, 31.25 mL of 2 M acetate buffer (pH 3.8), 12 mL acetic acid and water-saturated phenol stabilized (pH 4), at a final volume of 500 mL] was added, followed by incubation on a horizontal rotator (150 rpm, 10 min, RT). The liquid phase was transferred into a 2-mL microcentrifuge tube, was clarified by centrifugation (21,000×*g*, 5 min, 4 °C) and 1.6 mL of the supernatant were transferred to a new 2-mL tube, wherein 200 μL chloroform-isoamyl alcohol (24:1) were added, followed by vigorous shaking for 30 s, incubation (− 20 °C, 30 min) and centrifugation (21,000×*g*, 10 min, 4 °C). The upper aqueous phase (800 μL) was transferred and mixed with 667 μL isopropanol and 20 μL of magnetic beads (IDEXX Water DNA/RNA Magnetic Bead Kit; IDEXX Laboratories Inc., Westbrook, ME, USA), followed by incubation on a horizontal rotator (150 rpm, 15 min, RT). The beads were washed according to the manufacturer’s protocol. RNA was eluted in 100 μL buffer, and eluates were subjected to filtration, using the OneStep PCR Inhibitor Removal Kit (Zymo Research Corporation, Irvine, CA, USA) and were stored at − 80 °C. Extracted RNAs originating from 12 processed electronegative membranes and spanning 6 different days of sampling were pooled (1.1 mL total RNA extract) and mixed with 2.2 mL binding buffer containing isopropanol (IDEXX Water DNA/RNA Magnetic Bead Kit). Half of the mixture (1.65 mL) was incubated with 20 μL of magnetic beads on a horizontal rotator (150 rpm, 15 min, RT). After the magnetic separation of beads, the supernatant was removed and the procedure was repeated by adding the remaining 1.65 mL of the mixture. The beads were washed according to the manufacturer’s protocol and RNA was eluted in 60 μL buffer.

Concentrated RNAs were subjected to real-time RT-PCR testing for SARS-CoV-2 quantification, utilizing the N2 protocol proposed by the Centers for Disease Control and Prevention (CDC) for the diagnosis of COVID-19 in humans (CDC, 2020). The assay was performed on a CFX96 Touch Real-Time PCR Detection System (Bio-Rad Laboratories, Hercules, CA, USA). Calibration curves were generated using the synthetic single-stranded RNA standard “EURM-019” (Joint Research Centre, European Commission) and SARS-CoV-2 viral loads were expressed as genome copies per μL of RNA extract.

### Library preparation and sequencing

The targeted sequencing method was applied by preparing 400 nt amplicons using the ARTIC v3 protocol developed by Wellcome Sanger Institute^18^, with some modifications. First, cDNA synthesis was prepared from 10 μL of RNA using Super Script II reverse transcriptase (Invitrogen—Thermo Fisher Scientific, USA) and 50 ng/μL of random primers according to the protocol guidelines. For subsequent cDNA amplification, 2.5 μL of the generated cDNA was used instead of 6 μL, using ARTIC PCR primer pools (v3). Depending on the sequencing platform, libraries were submitted or not to the FS DNA Workflow for Illumina by a fragmentation step for 30 min at 37 °C, using the NEBNext Ultra II FS reagents (NEB#7658). Finally, the NEBNext adaptor (New England Biolabs, US, #7335) was used in the ligation reaction, diluted with adaptor dilution buffer at 10 μΜ final concentration. All purification steps were performed according to the ARTIC protocol. The samples were paired‐end, sequenced either on a MiSeq or a NextSeq500 platform (Illumina, USA) with a read length of 2 × 300 and 2 × 150 bp, respectively.

Library construction and sequencing was performed at the premises of CERTH INAB. The diagnostic facility at CERTH is an ISO 15189 certified unit. All steps are performed in dedicated physically separated areas with the appropriate measures. All the appropriate controls, both negative and positive ones according to Good Laboratory Practices and the adopted SOPs, have been used during the entire process.

### Raw data analysis

The initial phase of the bioinformatics analysis is to produce an alignment of the sequencing reads, while maintaining extremely strict criteria, in order to remove any potential contaminants and/or sequencing errors. The first step is the adaptor removal process, where any adaptors were removed from the raw *fastq* sequences, with the cleaned reads mapped to the SARS-CoV-2 reference genome (Wuhan variant, NC_045512), using Minimap2 tool^19^ with a minimal chaining score (matching bases minus log gap penalty) equal to 40. From this process, only the paired-end sequences were retained, while any other (unmatched, multiple mappings, etc.) were removed. In the next step, two different computational workflows were employed corresponding to the two different sequence lengths of 150-base or 300-base paired-end, used depending on the NGS platform. For the first seven samples and the last three (2 December 2020–18 March 2021 and 16 April 2021–06 May 2021), the primer sequences are excluded using the *iVar* tool^20^, setting a minimum of 200 length in nucleotides for a read to be retained after trimming, and a minimum threshold for sliding window of 15 quality to pass (width of sliding window equal to 4). The final sequences are then remapped to the same reference genome (minimal chaining score equal to 40). For the four samples between 19 March 2021 and 15 April 2021 primer trimming and remapping to reference genome was not applied, as the updated protocol used did not necessitate this step. Finally, in order to be able to detect low frequency mutations, the *freebayes* variant caller was used with a low mutation frequency parameter of 0.01. Ultimately, all identified mutations were annotated using the *SnpEff* tool^21^ and the NC_045512.2 (version 5.0) database. The proposed variant calling pipeline (with all set parameters) is provided on the GitHub repository.

### Downstream analysis of lineages detection

In order to identify and assign different SARS-CoV-2 lineages based on the mutations detected from a single wastewater sample, we implemented the proposed methodology in a tool named **lineagespot**.

**Figure 5:**
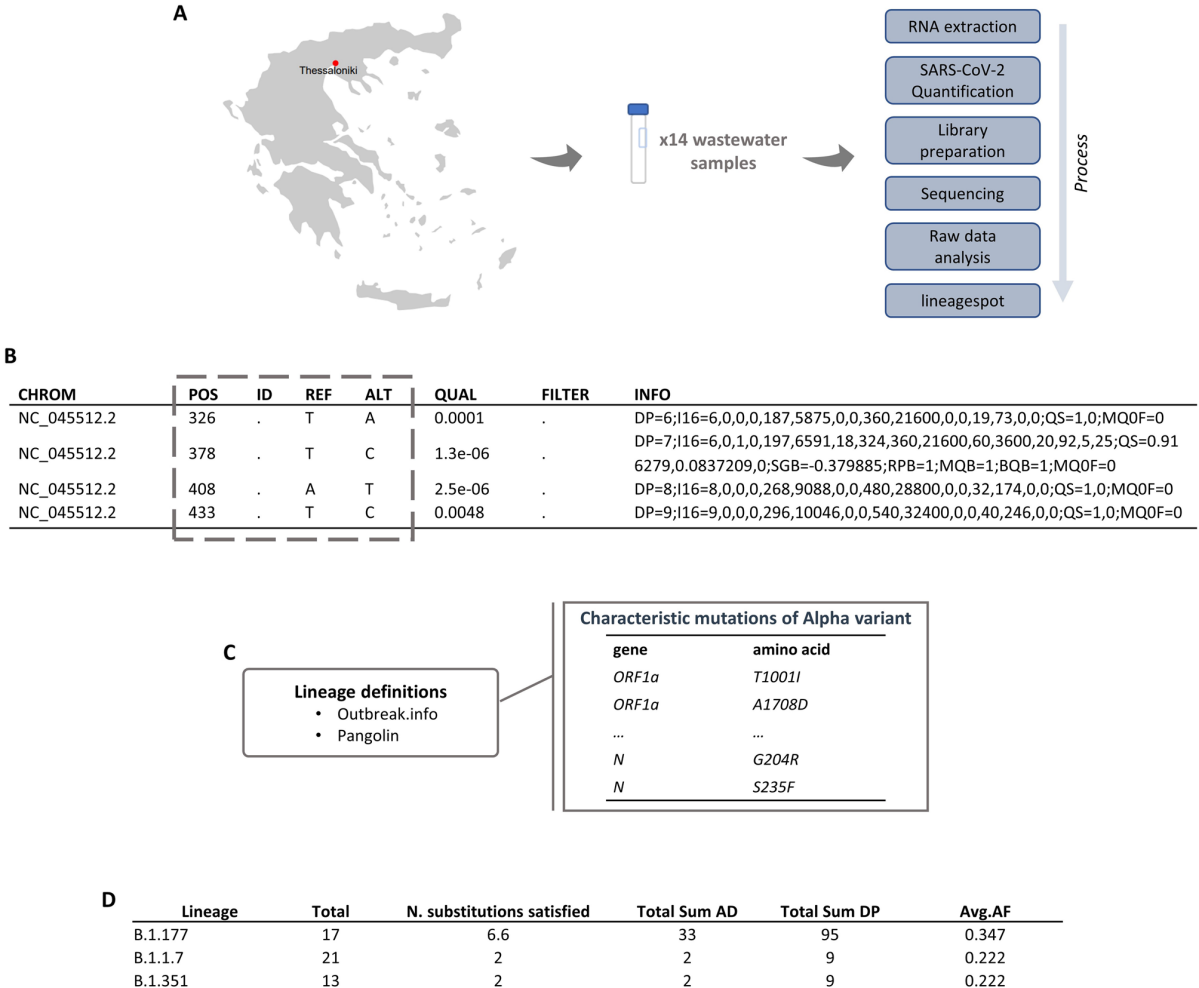
Snapshots of the intermediate steps. (**A**) A summary plot showing the overall process from the sampling to ***lineagespot.*** (**B**) A VCF file produced by the chosen variant caller (**C**) SARS-CoV-2 characteristic substitutions retrieved either from a public source (such as Pangolin or outbreak.info), or be user-provided. (**D**). A tab-delimited file as produced by ***lineagespot***.

The tool accepts as input a VCF file, which contains all nucleotide (and the corresponding amino acid) mutations identified in a sample, along with a file containing all lineage-assignment mutations. After analyzing all inputs, a tab-delimited file (TSV file) is produced containing the identified mutations that are related to SARS-CoV-2 lineages. In addition, we compute average allele frequencies to quantify lineage abundance metrics. Figure 5 shows an overview of the tool's functionalities.

**lineagespot** is dependent on the source used for retrieving lineage definitions. In our case, the two potential sources are (1) lineage-characteristic mutation profiles pre-calculated by outbreak.info and (2) lineage-characteristic mutation profiles derived from the trained Pangolin models. Both outbreak.info and Pangolin are available resources (https://cov-lineages.org/resources.html) for monitoring the SARS-CoV-2 outbreak, and have their data provided from GISAID. In this section we will describe the process for both cases; however, given the fact that the Pangolin definitions are based on a trained machine learning model that is not necessarily interpretable, **lineagespot** is using by default the first option (i.e. outbreak.info as the source of definitions):

i. Outbreak.info as the source of lineage definitions
  By default, **lineagespot** uses outbreak.info to retrieve information about lineage assignments. In order to do this, either an API is used, from which information regarding variants of concern or user specified variants and the related amino acid substitutions are retrieved, or user provided tab-delimited files can be given as an input to the tool in case a user is interested in specific mutations or lineages. Based on these inputs **lineagespot** identifies potential SARS-CoV-2 variants in the input samples and produces metrics to quantify the presence of each lineage.
ii. Pangolin as the source of definitions
  Initially, a vector of all genome positions is created, for which each position is set to be equal to the reference genome nucleotides. Then, every VCF file is read and a set of new rules is formed by setting each position of the file with the reported mutation or multiple mutations (in case there is more than one reported mutation at the same position). It should be noted that positions that have been detected with more than one mutation, should include all of them at the VCF’s ALT column in a comma-delimited format. Most of the variant caller tools (*freebayes*, *GATK*, etc.) are doing this by default. Finally, positions with reference read depth equal to zero are removed from the first vector. The remaining two vectors are merged into one. In addition to finding all positions that need to be set equal to the base that has been allocated, four more rules are added for each genome position. These rules contain all bases *not* equal to the nucleotide of the original rule. For example, if position 5388 is equal to base ‘A’ (representing rule *5388* =  = *‘A’*), then four new rules are added containing all bases not equal to ‘A’, e.g., *5888!* = *‘T’, 5388!* = *‘G’, 5388!* = *‘C’, 5388!* = *‘–’* (where the *‘–’* symbol stands for a gap in the referred sequence). Finally, all rules are merged into a single vector representing this particular lineage.

The second phase aims to compare the mutations derived from the VCF file with a lineage’s mutations. Specifically, all decision rules are read from the input Pangolin file, and for each lineage, the related rules are compared with the final rule vector.

It should be noted that having pangolin as a source of lineages’ definitions relies on the following assumption. Given a group of reads that satisfy a rule A of lineage L, and another group of reads that satisfy rule B from the same lineage L, then the lineage L is incorrectly assigned. As an example, suppose that a group of reads satisfy only the first two rules from lineage B.1.177.17 (28,931! = 'A', 25,613! = 'G'), and another group of reads satisfy the next two rules from the same lineage (407! = ‘–’, 22,087! = 'A'). Based on the method description above, lineage B.1.177.17 will be marked as an identified lineage, even though none of the reads satisfy all of the lineage’s rules. In order to mitigate this risk, we are taking into consideration a number of different indicators that reflect the number of total rules satisfied, the number of rules that are satisfied based on the detected mutations, and the overall number of reads that support both reference and allele for each of the rules. Three metrics are computed and stored in the output file; the total number of rules leading to the related lineage, the number of rules satisfied by the created rule vector, considering pangolin’s rules as a decision tree, and the total number of rules satisfied. Also, the related ratio values are being computed, giving a satisfaction percentage of each lineage.

### Source of lineage-specific mutations

For the detection of lineage-specific mutations, three different data sources were used; pangolin, VEO and *outbreak.info*. An example of the differences of the three sources is provided in Supplementary Fig. 1, which compares the detection of B.1.1.7 using (A) outbreak.info, (B) pangolin and (C) VEO data. **lineagespot** uses by default outbreak.info reports.

### Abbreviation matrix

See Table 7.

**Table 7:** Abbreviation table containing all acronyms that are used in the text.

Acronym/phrase	Meaning
*VoC*	Variant of concern
*NGS*	Next-generation sequencing
*VCF*	Variant call format
*CHROM*	The name of the sequence (typically a chromosome) on which the variation is being called
*POS*	The 1-based position of the variation on the given sequence
*REF*	The reference base (or bases in the case of an indel) at the given position on the given reference sequence
*ALT*	The list of alternative alleles at this position
*DP*	Read depth
*AD*	Read depth for each allele
*AF*	Allele frequency for each ALT allele in the same order as listed (use this when estimated from primary data, not called genotypes)
*HGVS*	Corresponding amino acid substitution
*N*_*t*_	Referring to nucleotide mutations

## Supplementary Information

Supplementary Figure 1.

Supplementary Tables.

Supplementary Information 3.
