# Supplementary material for: Detecting SARS-CoV-2 lineages and mutational load in municipal wastewater and a use-case in the metropolitan area of Thessaloniki, Greece
Source: Sci Rep. 2022 Feb 17;12:2659. doi: 10.1038/s41598-022-06625-6 (PMC8854625; doi:10.1038/s41598-022-06625-6)
Supplement: Supplementary file 1 — Supplementary Figure 1. [file 41598_2022_6625_MOESM1_ESM.docx]

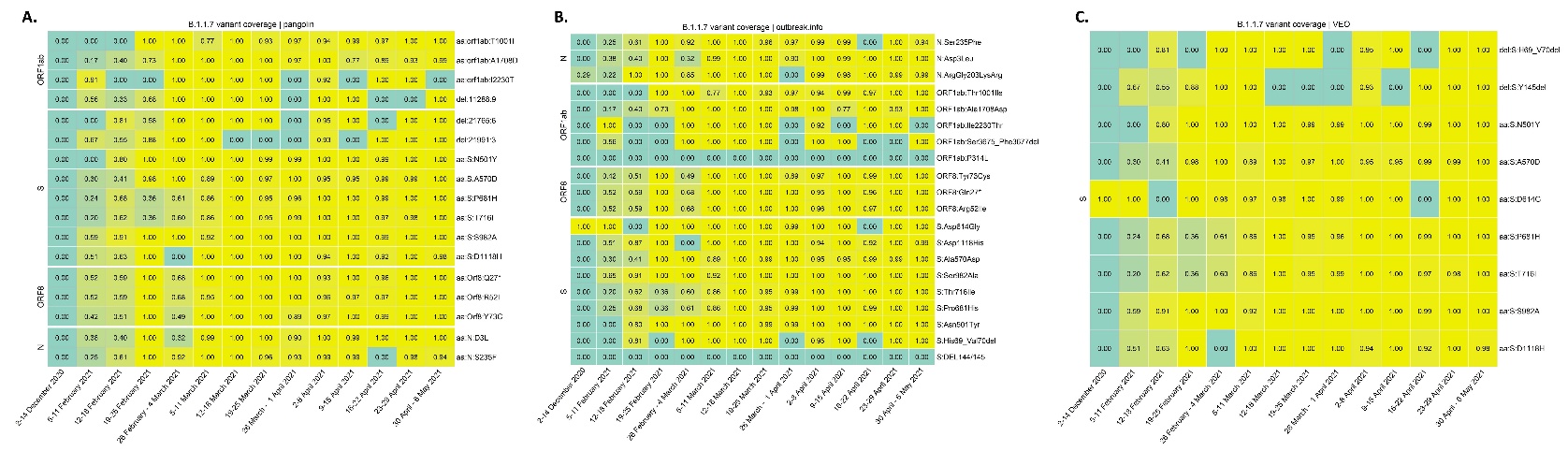


**Supplementary Figure 1:** Detected mutations of the Alpha variant (B.1.1.7) using data provided by **A.** outbreak.info, **B.** pangolin and **C.** VEO
