# Supplementary material for: Detecting SARS-CoV-2 lineages and mutational load in municipal wastewater and a use-case in the metropolitan area of Thessaloniki, Greece
Source: Sci Rep. 2022 Feb 17;12:2659. doi: 10.1038/s41598-022-06625-6 (PMC8854625; doi:10.1038/s41598-022-06625-6)
Supplement: Supplementary file 3 — Supplementary Information 3. [file 41598_2022_6625_MOESM3_ESM.docx]

# Supplementary Information

## **S1: Code and data**

The implemented code that produces the results of this paper, starting from the VCF files, is available on the GitHub repository: <https://github.com/BiodataAnalysisGroup/lineagespot>.

All raw FASTQ files are deposited on ENA: Project IDs **PRJEB44141** (*for patient samples*) and **PRJEB44548** (*for wastewater samples*).
